# Characterizing Glycosylation of Adeno-Associated Virus Serotype 9 Capsid Proteins Generated from HEK293 Cells through Glycopeptide Mapping and Released Glycan Analysis

**DOI:** 10.3390/microorganisms12050946

**Published:** 2024-05-07

**Authors:** Yu Zhou, Sonal Priya, Joseph Y. Ong

**Affiliations:** Analytical Development & Operations, Novartis Pharmaceuticals, 10210 Campus Point Drive, San Diego, CA 92121, USA; sonal.priya2693@gmail.com (S.P.); joseph.ong@novartis.com (J.Y.O.)

**Keywords:** AAV, capsid proteins, cIEF, glycosylation, glycopeptide mapping, LC-MS, LC-fluorescence detection

## Abstract

Recombinant adeno-associated viral (AAV) vectors have emerged as prominent gene delivery vehicles for gene therapy. AAV capsid proteins determine tissue specificity and immunogenicity and play important roles in receptor binding, the escape of the virus from the endosome, and the transport of the viral DNA to the nuclei of target cells. Therefore, the comprehensive characterization of AAV capsid proteins is necessary for a better understanding of the vector assembly, stability, and transduction efficiency of AAV gene therapies. Glycosylation is one of the most common post-translational modifications (PTMs) and may affect the tissue tropism of AAV gene therapy. However, there are few studies on the characterization of the N- and O-glycosylation of AAV capsid proteins. In this study, we identified the N- and O-glycosylation sites and forms of AAV9 capsid proteins generated from HEK293 cells using liquid chromatography–tandem mass spectrometry (LC-MS)-based glycopeptide mapping and identified free N-glycans released from AAV9 capsid proteins by PNGase F using hydrophilic interaction (HILIC) LC-MS and HILIC LC-fluorescence detection (FLD) methods. This study demonstrates that AAV9 capsids are sprinkled with sugars, including N- and O-glycans, albeit at low levels. It may provide valuable information for a better understanding of AAV capsids in supporting AAV-based gene therapy development.

## 1. Introduction

Adeno-associated virus (AAV) is a small non-enveloped virus with a single-stranded DNA genome that is encapsidated in an icosahedral protein capsid shell [1,2]. One AAV capsid consists of 60 viral proteins (VPs), including 5 VP1, 5 VP2, and 50 VP3. The three viral proteins share a common C-terminus and are present in one AAV at an approximate 1:1:10 ratio [3,4]. AAV vectors, with their nonpathogenic nature and the ability to provide long-term gene expression, have emerged as prominent gene delivery vehicles to treat a number of human diseases [5,6]. AAV capsid proteins determine tissue specificity and immunogenicity and play important roles in receptor binding, the escape of the virus from the endosome, and the transport of viral DNA to the nuclei of target cells. A recent study, by using a proximity ligation analysis, revealed that host factors, notably transcription-related machinery, are also selectively recruited by the VP1 N terminus of AAV8 [7]. The motifs for protein interaction, endosomal sorting, and signal transduction in eukaryotic cells have been identified on the VP1/2 N-terminus [8]. Furthermore, there are more than 13 AAV serotypes that show 51–99% capsid amino acid sequence identity [3,5,9].

The comprehensive characterization of AAV capsid proteins is necessary for a better understanding of the vector assembly, stability, and transduction efficiency of AAV gene therapies. A number of papers have addressed several common post-translational modifications (PTMs) of AAV capsid proteins, including deamidation [10,11,12,13,14,15,16,17], oxidation [13,14,15,16,17], and phosphorylation [13,14,16,17,18,19]. Only a few studies on the characterization of the N-glycosylation [13,20,21] and O-glycosylation [17] of AAV capsid proteins have been reported. However, these studies had different findings for the N-glycosylation of AAV capsids [13,20,21]. The N-glycosylation of AAV8 N^499^(NS) [20], AAV2 N^253^, AAV5 N^292^ and N^428^, AAV8 N^521^ and N^499^, and AAVrh10 N^306^ [13] was identified, but no glycosylation of AAV2 capsids prepared from cultured HeLa cells was reported [21]. 

For the identification of N- and O-glycosylation sites and forms of proteins, reversed-phase liquid chromatography–tandem mass spectrometry (RPLC-MS/MS)-based glycopeptide mapping is most widely used [22]. N-glycans can be released from glycoproteins by PNGase F, an amidase which cleaves between the innermost GlcNAc and asparagine residues of high mannose, hybrid, or complex oligosaccharides. After labeling the released N-glycans with fluorescent dyes, the N-glycans can then be analyzed by using hydrophilic interaction liquid chromatography–mass spectrometry (HILIC LC-MS), HILIC LC-UV, or HILIC LC-fluorescence detection (HILIC LC-FLD) [23]. In contrast to PNGase F, an enzyme which can remove almost all N-linked glycans from glycoproteins, there is no specific enzyme that can remove O-glycans.

The different findings [13,20,21] of the glycosylation of AAV capsid proteins may be due to the different cell lines and cell culture conditions used to generate recombinant AAV capsid proteins [24,25]. In addition, the sensitivities and resolutions of mass spectrometers, software, and glycan databases used for mass spec-based glycan analysis are critical for the detection and determination of glycosylation forms and sites. 

Glycosylation is one of the most common PTMs [26] and may affect the tissue tropism of AAV gene therapies [17,20]. Glycosylation occurs in eukaryotic and prokaryotic membranes and secreted proteins, and nearly 50% of plasma proteins are glycosylated [27]. Protein glycosylation has a well-established role in the folding, solubility, stability, binding, immunogenicity, and pharmacokinetics (PK) of proteins and biological therapeutics [24,25,28]. The presence of some glycans on proteins has been reported to play a role in virus entry and protection against neutralization, such as in the case of the envelope glycoprotein of hepatitis C virus (HCV) [29]. It was reported that mutations within potential glycosylation sites in the capsid protein of non-enveloped hepatitis E virus prevented the formation of infectious virus particles [30]. A high level of O-linked glycosylation of adenovirus type 5 fiber protein was also reported [31]. 

N-glycosylation occurs on the side chain of asparagine (N) residues within the consensus sequence motif, N-X-S/T (where N is asparagine, X is any amino acid except proline, S is serine, and T is threonine). There are five potential N-glycosylation sites on AAV9 capsid proteins, namely N^14^LS, N^262^ST, N^337^LT, N^452^GS, and N^497^NS. Among them, one site, N^14^LS, is located on the unique N-terminal region of VP1, while the other four sites are located on the shared regions of AAV9 VP1, VP2, and VP3. Here, we report the identification of the N- and O-glycosylation sites and forms of AAV9 capsid proteins generated from HEK293 cells using LC-MS/MS-based glycopeptide mapping and the identification of free N-glycans released from AAV capsid proteins by PNGase F using the HILIC LC-MS and HILIC LC-FLD methods. In addition, a capillary isoelectric focusing (cIEF) analysis was carried out to confirm the N-glycosylation of AAV9 capsids. This study demonstrates that AAV capsids are sprinkled with sugars, including N- and O-glycans with low occupancies. It may provide valuable information for a better understanding of AAV capsids in supporting AAV-based gene therapy development. 

## 2. Materials

AAV9 capsids used for glycosylation analysis were produced from HEK293 cells, purified by using chromatography and ultracentrifugation at Novartis Gene Therapies, and kept frozen at ≤−60 °C prior to use. Iodoacetamide (IAM), 1 M triethylammonium bicarbonate solution (TEAB), and 20% sodium dodecyl sulfate (SDS) solution in water were purchased from Sigma (St. Louis, MO, USA). Acetonitrile, 0.1% formic acid in acetonitrile, water, 0.1% formic acid in water, and methanol were LC-MS grade and obtained from Honeywell Burdick&Jackson (Muskegon, MI, USA). Phosphoric acid (85%), 0.5 M tris (2-carboxyethyl) phosphine (TCEP), LC-MS grade formic acid, and LC-MS grade trifluoroacetic acid were from Thermo Fisher Scientific (Rockford, IL, USA). Trypsin/LysC Mix Mass Spec Grade was purchased from Promega (Madison, WI, USA). S-Trap columns were from ProtiFi (Huntington, NY, USA).

Human IgG1 standard (Umab1) was purchased from GlycoScientific, LLC (Athens, GA, USA). The Waters GlycoWorks RapiFluor-MS N-Glycan—24 Sample Kit, including RapiGest surfactant, Rapid PNGase F enzyme, anhydrous N, N-dimethylformamide (DMF), HILIC μElution plate, SPE Elution Buffer (200 mM ammonium acetate in 5% acetonitrile), and mouse IgG1 standard, was purchased from Waters (Milford, MA, USA). The 96-well Plate Extraction Vacuum Manifold and Waters Acquity UPLC Glycan BEH Amide column (130 Å, 1.7 μm, 2.1 × 150 mm) were also purchased from Waters. The Halo Penta-HILIC column (90 Å, 2.7 μm, 2.1 × 150 mm) was purchased from Advanced Materials Technology (Wilmington, DE, USA). Amicon 50 kDa centrifugal filters were purchased from Merck Millipore Ltd. (Burlington, MA, USA). SpeedVac Vacuum Concentrator was purchased from Thermo Scientific (Waltham, MA, USA). The software Skyline (Version 21.2.0.536) from the MacCoss Lab at the University of Washington was utilized for released glycan mass spec data analysis.

For capillary isoelectric focusing (cIEF) analysis, the cIEF instrument, Maurice Capillary Electrophoresis Analyzer with Compass software from ProteinSimple (San Jose, CA, USA), was used. Maurice cIEF cartridges, glass reagent vials and appropriate caps, a 96-well plate, anolyte (80 mM phosphoric acid in 0.1% methyl cellulose), catholyte (100 mM sodium hydroxide in 0.1% methyl cellulose), fluorescence calibration solution, 0.5% and 1% methyl cellulose, 500 mM arginine, pI markers (at pH 4.05 and 9.50) prepared in DI water according to the manufacturer’s instructions, and Pharmalyte carrier ampholytes were also from ProteinSimple (San Jose, CA, USA). Dimethyl sulfoxide (DMSO) was purchased from JT Baker (Phillipsburg, NJ, USA). Formamide was purchased from Acros Organics (Thermo Fisher Scientific, now). PNGase F was purchased from New England BioLabs (Ipswich, MA, USA).

## 3. Methods

### 3.1. Glycopeptide Mapping

1. Sample preparation for glycopeptide mapping

The denaturation and reduction of AAV9 capsid proteins were carried out in 1% SDS buffer and 10 mM TCEP at 60 °C for 10 min, respectively. After the protein solution was cooled down to room temperature (RT), iodoacetamide (IAM) was added in the sample at the final concentration of 10 mM for alkylation at RT for 30 min in the dark. The sample was acidified by adding 12% aqueous phosphoric acid at a ratio of 1:10 *v*/*v* for a final concentration of ~1.2% to pH ~ 1. The acidified SDS sample was diluted 9-fold using an S-Trap binding buffer, and the mixture of the acidified SDS sample and the S-Trap binding buffer was loaded on an S-Trap column. The S-Trap protocol provided by ProtiFi was followed to wash the column with the S-Trap binding buffer, and then the proteins were digested with trypsin at a ratio of 1:20 *w*/*w* of enzyme to protein at 37 °C for 1 h. The tryptic peptides were first eluted with 0.2% aqueous formic acid and then with 50% acetonitrile containing 0.2% formic acid to recover all peptides.

2. Reversed-phase liquid chromatography–tandem mass spectrometry (LC-MS/MS)

The reversed-phase liquid chromatography–tandem-mass spectrometry (LC-MS/MS) system used for glycopeptide mapping was a Thermo Scientific^TM^ ULtiMate^TM^ 3000 RSLC nano system (Thermo Fisher Scientific) coupled with a Thermo Scientific^TM^ Orbitrap Eclipse™ Tribrid™ Mass Spectrometer (Thermo Fisher Scientific). AAV9 capsid tryptic peptides were separated on an EASY-Spray™ column, PepMap RSLC C18 (3 μm, 75 μm × 150 mm, Thermo Fisher Scientific) at a column temperature of 35 °C using 0.1% formic acid in water as mobile phase A and 0.1% formic acid in acetonitrile as mobile phase B at a flow rate of 300 nL/min. The peptides were eluted with a gradient from 2 to 10% mobile phase B over 2 min, then 10–20% mobile phase B over 40 min, 20–40% mobile phase B over 12 min, 40–75% mobile phase B over 8 min, and then an isocratic hold at 75% mobile phase B for 5 min before changing mobile phase B back to 2%. 

The mass spectrometry data were acquired using a top 10 data-dependent acquisition (DDA) method on the mass spectrometer fitted with a nanospray ionization source (EASY-Spray Source) (Thermo Fisher Scientific). The following mass spectrometer parameters for glycopeptide mapping were used: the static spray voltage was set at 3000 V for the positive ion polarity mode, and the ion transfer tube temperature was set to 300 °C. The Orbitrap mass analyzer was used for MS full survey scan (*m*/*z* = 200–2000) at a resolution of 120,000 at 200 *m*/*z*, the S-lens radio frequency (RF) level was set at 30%, the AGC Target was set to standard, and the maximum injection time mode was set to auto. The quadrupole mass analyzer was used for MS^2^ scans using higher energy collision dissociation (HCD) at a collision energy of 30, with the isolation window of 0.7 *m*/*z*. The resolution for HCD spectra was set to 60,000 at 200 *m*/*z*. Peptides of charge states 2–7 were selected with a signal intensity threshold of 1 × 10^4^. Precursor ions with single, unassigned, or seven and higher charge states were excluded. The method also used dynamic exclusion with a 20 s exclusion duration and the mass tolerance was set to 10 ppm.

3. Mass spectra data analysis

The Byos^®^ software (v4.6, Protein Metrics Inc., Boston, MA, USA) was used for MS/MS data analysis to determine N- and O-glycosylation sites and forms. The AAV9 VP1 sequence (Uniprot Q6JC40) was used for performing sequence matching. A database containing 132 human N-glycans and a database containing the six most common O-glycans, including HexNAc(1), HexNAc(2), HexNAc(1)Hex(1), HexNAc(2)Hex(1), HexNAc(1)Hex(1)NeuAc(1), and HexNAc(1)Hex(1)-NeuAc(2), were used in the software for the identification of N-glycans and O-glycans, respectively. Parameters applied for sequence matching included the following: carbamidomethylation (+57.021464 Da) set as a fixed modification, and N-terminal initial methionine residue loss followed by acetylation of alanine of AAV9 VP1 (−89.02992 Da), deamidation (+0.984016 Da), and oxidation (+15.994915 Da) set as variable modifications. The precursor mass tolerance was set to 10 ppm, and the fragment mass tolerance was set to 15 ppm. These data were validated at a 1% false discovery rate using standard reverse-decoy techniques. Peptide MS/MS spectra match ≥2 was set as acceptance criteria for peptide identification to assure the accuracy and confidence of peptide identification and assignment. The peptide spectra and all PTM mass spectra were also manually validated to confirm identification. 

### 3.2. Released free Glycan Analysis

1. Deglycosylation and labeling of released N-glycans

The AAV9 sample was concentrated using an Amicon 50 kDa centrifugal filter to reach a protein concentration of 5.2 μg/μL. TCEP in LC-MS grade water and 5% (*w*/*v*) RapiGest surfactant were added to the concentrated sample to a final concentration of 4 mM TCEP and a final protein concentration of ~1.35 μg/μL. The sample was then subjected to protein denaturation by heat at 75 °C for 3 min. After the sample tube was cooled down to RT, N-glycans were released from proteins by GlycoWorks Rapid PNGase F enzyme with incubation of the sample tube at 50 °C for 5 min. The deglycosylation mixture was then subjected to labeling with the Waters RapiFluor MS labeling kit.

The user manual was followed when using the Waters RapiFluor MS labeling reagent.

Briefly, one vial containing 9 mg of labeling reagent was mixed with 131 μL of anhydrous N, N-dimethylformamide (DMF). The prepared RapiFluor MS reagent solution (12 μL) was then added to the deglycosylation mixture. The labeling reaction was carried out for 5 min at RT. The sample clean-up was performed using Waters GlycoWorks HILIC μElution plate fitted to a vacuum manifold. The labeled sample solution prepared above was diluted with acetonitrile, and the diluted sample was loaded on a well of the μElution plate that was washed with LC-MS grade water and equilibrated with the same volume (200 µL) of water/acetonitrile in a ratio of 15:85. After washing the well with 600 μL of formic acid/water/acetonitrile at a ratio of 1:9:90 (*v*/*v*/*v*) twice, the N-glycans were then eluted with 30 μL of GlycoWorks SPE Elution Buffer (200 mM ammonium acetate in 5% acetonitrile) three times. The RapiFluor MS (RFMS) labeled N-glycan solution (90 μL) was concentrated to ~10 μL using a SpeedVac (Thermo Scientific) and diluted with equal volumes of GlycoWorks Sample Diluent–N-dimethylformamide/acetonitrile (50:50) prior to HILIC LC-FLD (fluorescence detection) and HILIC LC-MS analyses. 

The deglycosylation and labeling of the free N-glycans released from two standards, mouse IgG1 and human IgG1 monoclonal antibodies (mAbs), were performed using the same protocol as the one used for AAV9 capsid proteins.

2. Hydrophilic interaction liquid chromatography-fluorescence detection (HILIC LC-FLD) and LC-MS for analysis of released labeled N-glycans

The hydrophilic interaction liquid chromatography-fluorescence detection (HILIC LC-FLD) using a Waters Acquity UPLC coupled to a fluorescence detector (FLD) and the LC-MS using a Thermo Scientific Vanquish UHPLC coupled to an Orbitrap Eclipse™ Tribrid™ mass spectrometer were used for the data acquisition of the released labeled N-glycans. For LC-FLD data acquisition, the labeled N-glycans were separated on a Waters Acquity UPLC Glycan BEH Amide column (130 Å, 1.7 μm, 2.1 × 150 mm) using 50 mM ammonium formate in LC-MS grade water as mobile phase A and 100% acetonitrile as mobile phase B. For LC-MS data acquisition, the labeled N-glycans were separated on an Advanced Materials Technology Halo Penta-HILIC column (90 Å, 2.7 μm, 2.1 × 150 mm) using 50 mM ammonium formate with 0.1% formic acid in LC-MS grade water as mobile phase A and 0.1% formic acid in acetonitrile as mobile phase B. The optimized LC gradient used for the analysis of the released labeled N-glycans was 80% acetonitrile held for 8 min, 80% to 50% acetonitrile ramped over 30 min, 10% acetonitrile held for 10 min to wash the column, and 80% acetonitrile held for 10 min to equilibrate the column for the next run. The flow rate was set to 0.2 mL/min. Column temperature was set to 60 °C. The excitation and emission wavelengths were set to 265 nm and 425 nm for RapiFluor-MS label.

3. Data analysis for released labeled N-glycans

The Skyline software (Version 23.1) under the molecule interface mode was used for the determination of released free glycans. The mass spec raw data files of three replicates were imported to Skyline, and the data analysis was performed by checking the same database containing 132 human N-glycans, which was used in the glycopeptide mapping. Mass error and peak intensities were reported as recorded in Skyline. 

### 3.3. Comparison of AAV9 Capsids Treated with and without PNGase F Using Capillary Isoelectric Focusing (cIEF) 

AAV9 capsids were treated with PNGase F in GlycoBuffer 2 or enzyme buffer GlycoBuffer 2 (New England Biolabs) only as a control at 37 °C for 1 h. The samples were then denatured and reduced using TCEP at a final concentration of 20 mM in 50% (*v*/*v*) dimethyl sulfoxide (DMSO) at 70 °C for 10 min. The entire volume of the above prepared samples was used for cIEF analysis as described in [32], except the concentration of formamide was decreased to 40% by volume to increase sample concentration. The fluorescence detection default setting was excitation at 280 nm and emission at 458 ± 30 nm, and the exposure time used was 20 s. The samples were prepared independently across two different days (biological replicates); each biological replicate was injected either two or three times (each injection was considered a technical replicate). The data were analyzed in Compass and then exported to Chromeleon (Thermo Fisher Scientific) for better visualization. A standard IgG1 monoclonal antibody was used as a control and analyzed in the same way as the AAV9 capsids to ascertain the completion of deglycosylation.

## 4. Results 

### 4.1. Identification of N-Glycosylation Sites and Forms of AAV9 Capsids by Glycopeptide Mapping

**Figure 1:**
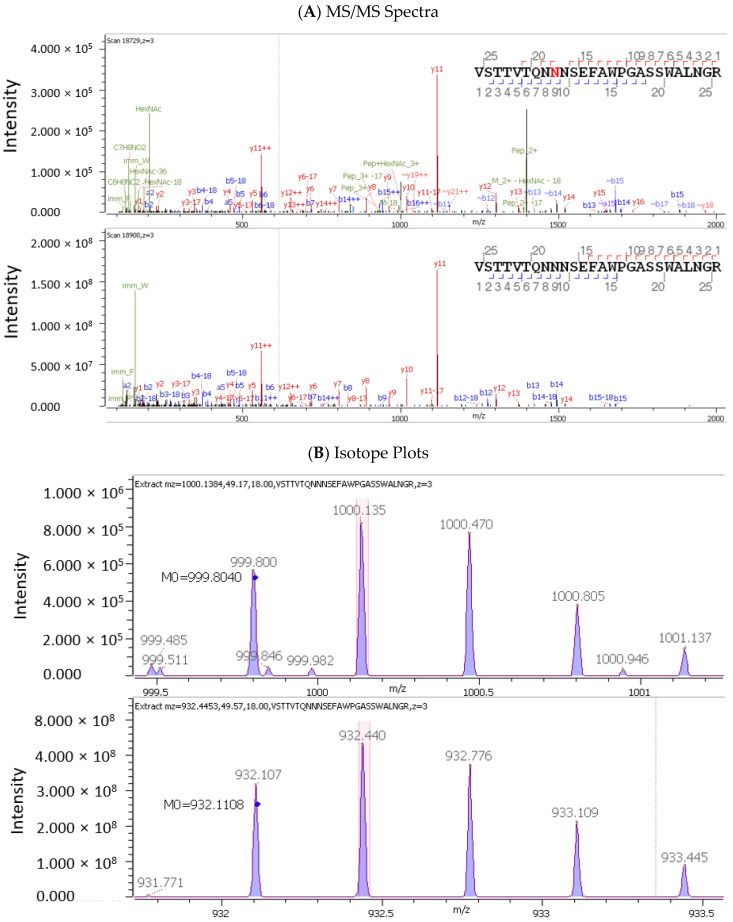

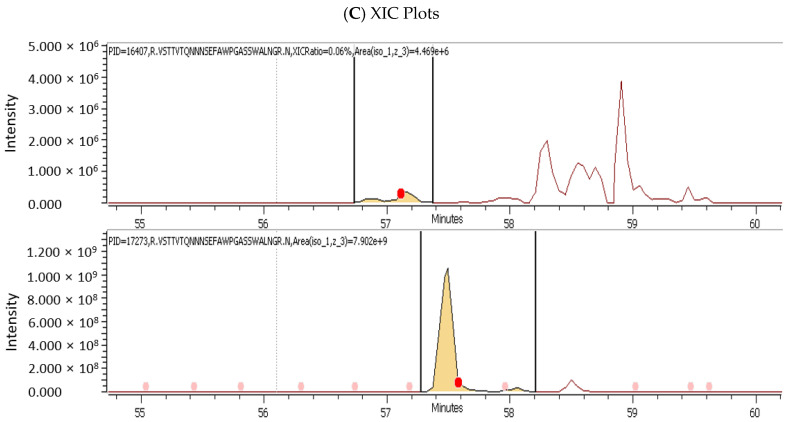
The identification of the N-glycan diagnostic ion HexNAc(1) at *m*/*z* of 203.0794 attached on N^497^ (NS) by glycopeptide mapping. (**A**) The MS/MS spectra of the tryptic peptide containing N^497^(NS) with HexNAc(1) attached (N^497^ in red) (upper trace) and the corresponding unglycosylated tryptic peptide (lower trace). (**B**) The upper trace represents the isotope plot of tryptic peptide containing N^497^(NS) with HexNAc(1) attached, and the lower trace represents the isotope plot of the corresponding unglycosylated tryptic peptide. (**C**) The upper trace represents the XIC plot of tryptic peptide containing N^497^(NS) with HexNAc(1) attached, and the lower trace represents the XIC plot of the corresponding unglycosylated tryptic peptide. The occupancy of N^497^(NS) with HexNAc(1) attached is 0.06%.

From the glycopeptide analysis of multiple lots of AAV9 capsids, the oxonium ion HexNAc(1) at *m*/*z* of 203.0794 was found attached on N^497^ of the tryptic peptide VSTTVTQN-N^497^NSEFAWPGASSWALNGR. This oxonium ion is a diagnostic ion that indicates the attachment of an N-glycan on N^497^ (NS) within the consensus sequence motif, N-X-S/T. The MS/MS spectra (Figure 1A) of the tryptic peptide containing N^497^(NS) with HexNAc(1) attached (upper trace) and the corresponding unglycosylated tryptic peptide (lower trace) and isotope plots (Figure 1B) confirm the glycosite of N^497^(NS). This result is in agreement with the previous observations of N-glycosylation on N^499^(NS) of AAV8 capsid proteins [13,20]. However, the occupancy of N-glycosylated N^497^(NS) of AAV9 capsids with HexNAc(1) attached was low (~0.06%) based on the comparison with the corresponding unglycosylated tryptic peptide containing N^497^(NS) (Figure 1C). 

While HexNAc(1) attached on N^497^(NS) was identified in all injections of the prepared AAV9 tryptic peptides, other N-glycoforms attached on N^497^(NS), more types of N-glycoforms on N^377^(LT), and fewer types of N-glycoforms on N^262^(ST) were identified in some but not all injections, indicating much lower occupancies than the occupancy of HexNAc(1) on N^497^(NS). Overall, the low occupancies of N-glycosylation on AAV9 capsids resulted in low mass spec signals. No N-glycosylation forms were identified on the potential N-glycosylation sites N^14^(LS) and N^452^(GS).

**Figure 2:**
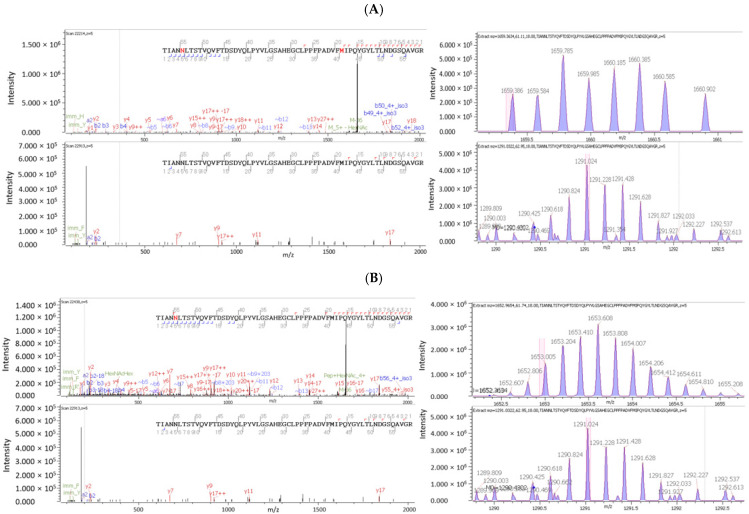
The identification of N-glycosylation on N^377^(LT) by glycopeptide mapping. (**A**) The MS/MS spectra (left) and isotope plots (right) of the tryptic peptides containing N^377^(LT) with the N-glycan HexNAc(5)Hex(5) at m/z of 1825.6610, z = 5, attached on N^377^(LT). (**B**) The MS/MS spectra (left) and isotope plots (right) of the tryptic peptides containing N^377^(LT) with N-glycan HexNAc(5)Hex(4)Fuc(1) at *m*/*z* of 1809.6661, z = 5, attached on N^377^(LT).

Figure 2 shows the MS/MS spectra and isotope plots of the tryptic peptide containing N^377^(LT) with the N-glycan HexNAc(5)Hex(5) at *m*/*z* of 1825.6610, z = 5 (Figure 2A), or HexNAc(5)Hex(4)Fuc(1) at *m*/*z* of 1809.6661, z = 5 (Figure 2B), attached on N^377^(LT). The isotope plot of the tryptic peptide containing N^377^(LT) with the N-glycan HexNAc(5)Hex(5) (upper trace) in Figure 2A and the isotope plot of the tryptic peptide containing N^377^(LT) with the N-glycan HexNAc(5)Hex(4)Fuc(1) (upper trace) in Figure 2B are different due to the different masses of these two N-glycans attached. Note that the isotope plots (lower traces) of the corresponding unglycosylated tryptic peptide in Figure 2A,B are identical because the corresponding unglycosylated tryptic peptide is the same. 

### 4.2. Identification of O-Glycosylation Sites and Forms of AAV9 Capsids by Glycopeptide Mapping 

Glycopeptide mapping also demonstrated the presence of multiple O-glycosylation forms on AAV9 capsids. Four of the six most common O-glycans were identified on the AAV9 capsids. The identified O-glycans and O-glycosylation sites are listed in Table 1. 

**Figure 3:**
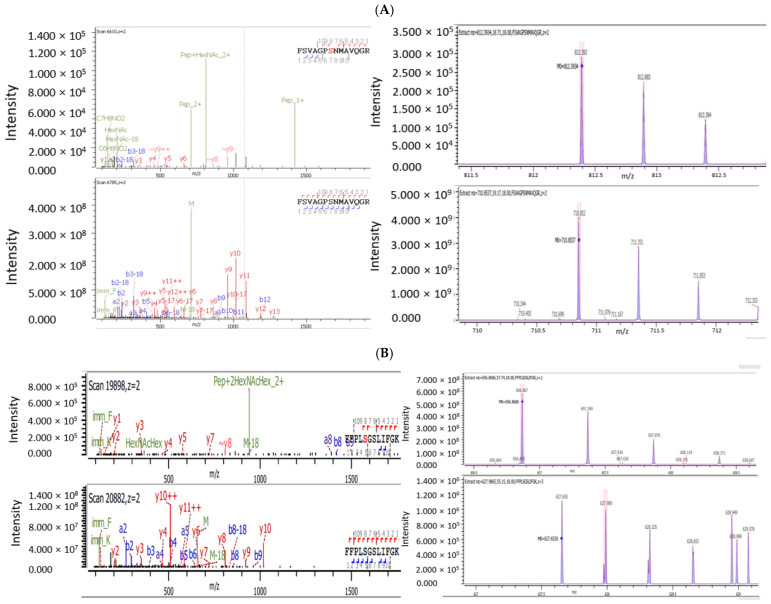
The identification of O-glycosylation on AAV9 capsids by glycopeptide mapping. (**A**) The MS/MS spectra (left) and isotope plots (right) of the tryptic peptides containing S^469^ with or without O-glycan HexNAc(1) at *m*/*z* of 203.0794 attached on S^469^. (**B**) The MS/MS spectra (left) and isotope plots (right) of the tryptic peptides containing S^538^ with or without O-glycan HexNAc(2)Hex(1) at *m*/*z* of 568.2116 attached on S^538^.

The O-glycan HexNAc(1) at *m*/*z* of 203.0794 was found attached on five serine residues, S^162^, S^469^, S^499^, S^586^, and S^632^, and one threonine residue, T^241^; HexNAc(2) at *m*/*z* of 406.1587 was found attached on S^348^; HexNAc(2)Hex(1) at *m*/*z* of 568.2116 was found attached on S^538^ and T^548^; and HexNAc(1)Hex(1)NeuAc(1) at *m*/*z* of 656.2276 was found attached on T^339^ and T^381^. The MS/MS spectra and isotope plots of the tryptic peptide containing O-glycans, namely HexNAc(1) attached on S^469^ and HexNAc(2)Hex(1) at *m*/*z* of 568.2116 attached on S^538^, are shown in Figure 3. Figure 3A shows the MS/MS spectra and isotope plots of the tryptic peptides containing S^469^ with or without the O-glycan HexNAc(1) at *m*/*z* of 203.0794 attached on S^469^. Two y ions, y8 and y9, containing S^469^ with HexNAc(1) attached (S^469^ in red) confirm the O-glycosylation. Figure 3B shows the MS/MS spectra and isotope plots of the tryptic peptides containing S^538^ with or without the O-glycan HexNAc(2)Hex(1) at *m*/*z* of 568.2116 attached on S^538^. Two b ions, b8 and b9, and one y ion, y8 containing S^538^ with HexNAc(2)Hex(1) attached (S^538^ in red), confirm the O-glycosylation.

### 4.3. Identification of Labeled Released N-Glycans from AAV9 Capsids 

**Figure 4:**
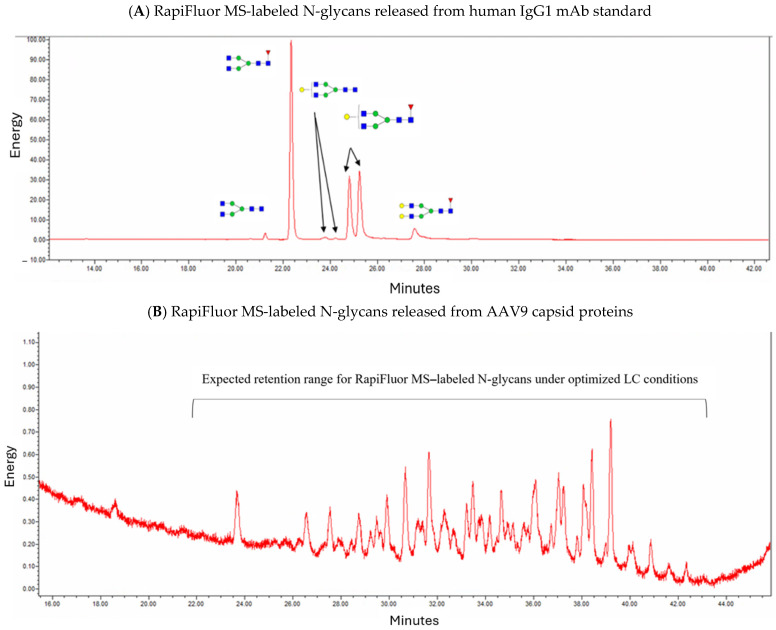
HILIC LC-FLD chromatograms of RapiFluor MS-labeled released free N-glycans from human IgG1 antibodies and AAV9 capsids. Mobile phase (**A**) 50 mM ammonium formate. Mobile phase (**B**) 100% acetonitrile. Column: Waters Acquity UPLC Glycan BEH Amide column. Column temperature: 60 °C.

To identify the labeled released N-glycans, the labeled released N-glycans were separated using hydrophilic interaction liquid chromatography (HILIC LC), which is commonly used to separate polar compounds that are not resolved in reversed-phase LC (RPLC). The more hydrophilic the N-glycan, the longer the retention on a HILIC column. The initial condition in the HILIC mode is highly organic, typically 80–90% acetonitrile, and the polar compounds are then eluted off the column by increasing the polarity of the mobile phase, such as by making it more aqueous. The optimization of the LC gradient was performed using the labeled released N-glycans from the IgG1 standards to ensure a good separation of labeled released N-glycans and appropriate retention times, specifically neither too early around the initial void volume peak nor too late, where the peak shapes and resolution may be compromised (Figure 4A). The labeled released N-glycans from the AAV9 capsids were run under the optimized gradient condition (Figure 4B). The peaks visualized by fluorescence detection in the expected range of 22–42 min indicated the possibility of the presence of N-glycans in the AAV9 samples, albeit at trance levels in comparison to the N-glycan levels of IgG1 (Figure 4A). 

**Figure 5:**
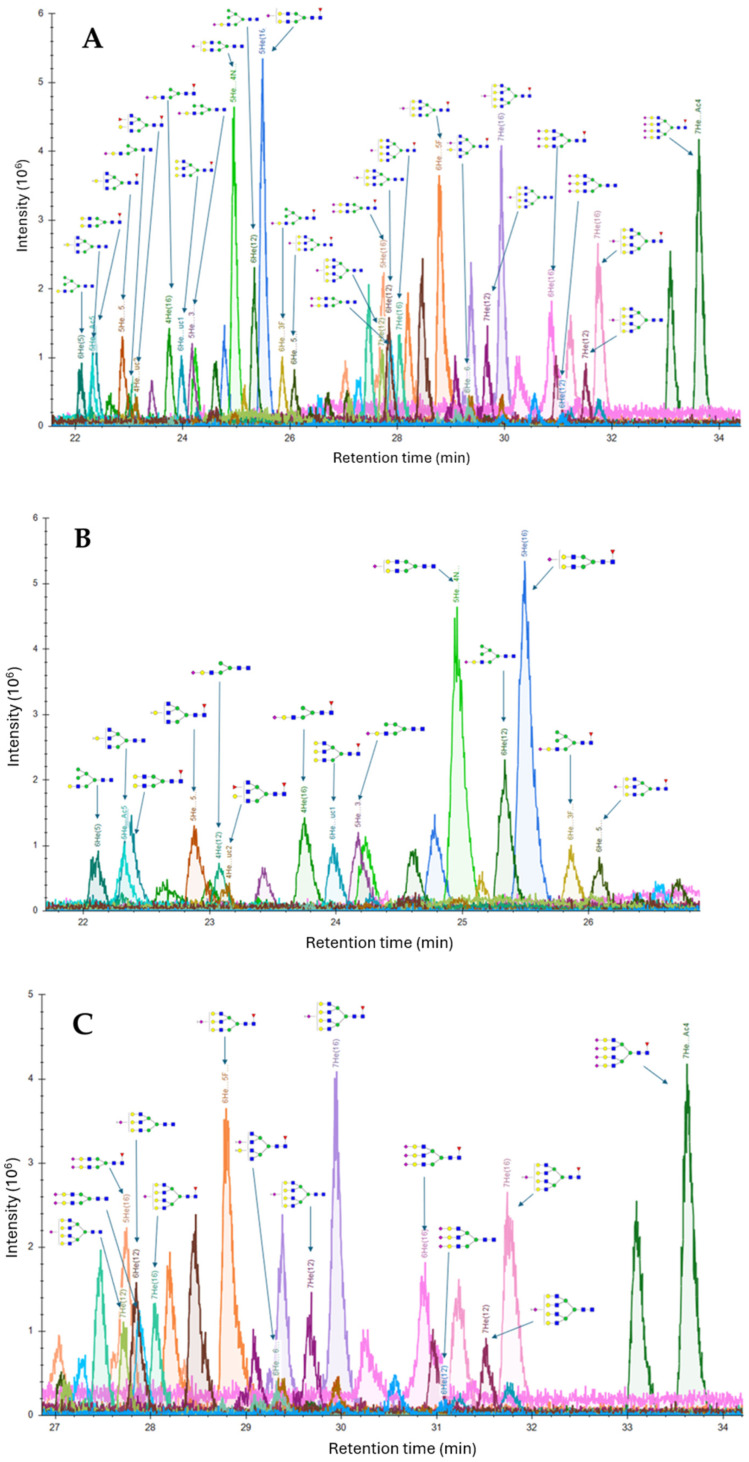
HILIC LC-MS chromatogram of RapiFluor MS-labeled N-glycans released from AAV9 capsids. (**A**) Full view of chromatogram. (**B**) Zoomed-in region of chromatogram at retention time of 22–26.8 min. (**C**) Zoomed-in region of chromatogram at retention time of 27–34 min. Mobile phase (**A**): 50 mM ammonium formate with 0.1% formic acid in LC-MS grade water. Mobile phase (**B**): 0.1% formic acid in acetonitrile. Column: Advanced Materials Technology Halo Penta-HILIC column. Column temperature: 60 °C.

To identify the labeled released N-glycans from the AAV9 capsids, a mass spec data analysis was carried out using the Skyline software and the same 132 human N-glycans database used for glycopeptide mapping. Twenty-eight (28) released N-glycans, including 4 hybrid and 24 complex N-glycans, were identified in the AAV9 sample (Figure 5). Figure 5A shows the full view of the chromatogram, and Figure 5B,C show the zoomed-in region of the chromatogram at the retention times of 22–26.8 min and 27–34 min, respectively. The identified labeled released N-glycans are listed in Table 2. The Byos software for released N-glycan analysis from Protein Metrics was used to compare the results from the Skyline software using the same database. Notably, the same 28 N-glycans identified using the Skyline software were also identified using the Byos software. 

**Figure 6:**
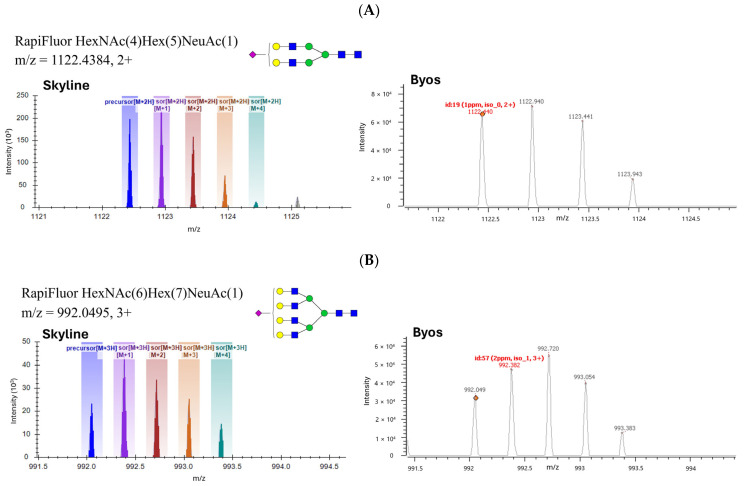

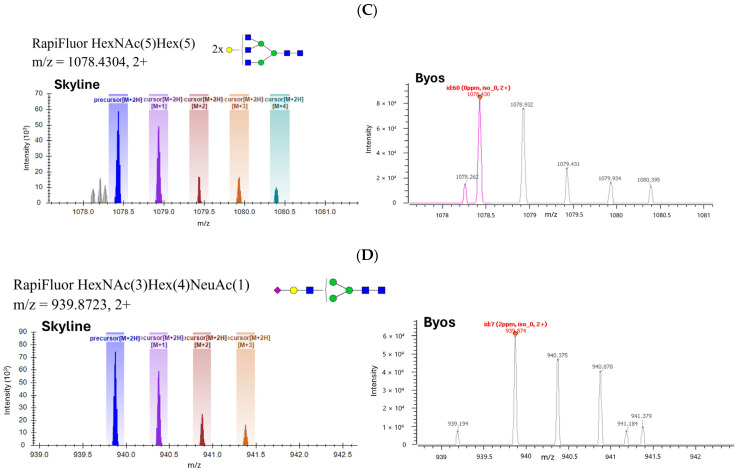
MS1 spectra of RapiFluor MS-labeled N-glycans released from AAV9 capsids. (**A**) MS1 spectra of RapiFluor MS-labeled HexNAc(4)Hex(5)NeuAc(1). (**B**) MS1 spectra of RapiFluor MS-labeled HexNAc(6)Hex(7)NeuAc(1). (**C**) MS1 spectra of RapiFluor MS-labeled HexNAc(5)Hex(5). (**D**) MS1 spectra of RapiFluor MS-labeled HexNAc(3)Hex(4)NeuAc(1).

Figure 6 shows the MS1 spectra and isotope plots of the N-glycans HexNAc(4) Hex(5)NeuAc(1), HexNAc(6)Hex(7)NeuAc(1), HexNAc(5)Hex(5), and HexNAc(3)-Hex(4)NeuAc(1) from the Skyline and Byos software, respectively. In addition to their MS1 spectra, the relative HILIC retention times were also considered for determining N-glycan identity. More hydrophilic and heavier N-glycans had longer retention times on HILIC compared to the less polar N-glycans. For instance, N-glycans containing sialic acids eluted later than the glycans without sialic acid. For example, HexNAc(4)Hex(5)Fuc(1) eluted at the retention time of 22.4 min, while HexNAc(4)Hex(5)Fuc(1)NeuAc(1) eluted later at the retention times of 24.8 and 25.5 min. The two chromatographic peaks for this N-glycan could be attributed to the 2,3 and 2,6-linkages of the sialic acids. The N-glycan with 2,3-linked sialic acid has been reported to be eluted before the 2,6-linked sialic acid on a HILIC column [33]. Similarly, the N-glycan HexNAc(4)Hex(5)Fuc(1)NeuAc(2) eluted later than HexNAc(4)Hex(5)Fuc(1)NeuAc(1) at the retention times of 27.0 min, 27.7 min, and 28.4 min, about 2.5 min later than HexNAc(4)Hex(5)Fuc(1)NeuAc(1). The first peak could correspond to a structure containing sialic acids with 2,3- and 2,3- linkages, respectively, the second peak could correspond to a mixture of 2,3- and 2,6 as well as 2,6- and 2,3- linked sialic acids, and the third peak could correspond to 2,6- and 2,6- linked sialic acids. The average increase in retention time upon adding sialic acid was 2.2 min under the LC-MS conditions. The approximately 2.5 min shift in retention time upon the addition of one sialic acid from HexNAc(4)Hex(5)Fuc(1)NeuAc(1) to HexNAc(4)Hex(5)Fuc(1)NeuAc(2) is consistent with the 2.2 min shift in retention time upon the addition of one sialic acid from HexNAc(4)Hex(5)Fuc(1) to HexNAc(4)Hex(5)Fuc(1)NeuAc(1).

Furthermore, there was an average increase of 0.5 min when a fucose was added to an N-glycan structure, such as in the case of HexNAc(3)Hex(4)NeuAc(1) at 23.1 min and HexNAc(3)Hex(4)Fuc(1)NeuAc(1) eluting at 23.7 min (a difference of 0.6 min). Moreover, the average retention shift upon the addition of a GlcNAc moiety resulted in an average retention increase of 0.5 min, as can be observed from the retention shift between HexNAc(4)Hex(5)Fuc(1) at 22.4 min and HexNAc(5)Hex(5)Fuc(1) at 22.9 min. Thus, in addition to the MS1 data, the relative retention time shifts in the HILIC mode were useful in assigning and confirming the identity of the released N-glycans. 

### 4.4. Confirmation of N-Glycosylation of AAV9 Capsids by Using Capillary Isoelectric Focusing (cIEF) 

Capillary isoelectric focusing electrophoresis (cIEF) is a technique that separates proteins according to their isoelectric points (pI) based on the charge properties of proteins and is widely used for the characterization of proteins. As an orthogonal method of verifying the presence of N-glycosylation on AAV9 capsids, cIEF was used to analyze AAV9 capsids with or without PNGase F treatment. During the deglycosylation reaction, PNGase F converts a glycosylated asparagine residue into an aspartic acid residue. This enzymatic deamidation can be detected by cIEF, as it introduces a negative charge.

**Figure 7:**
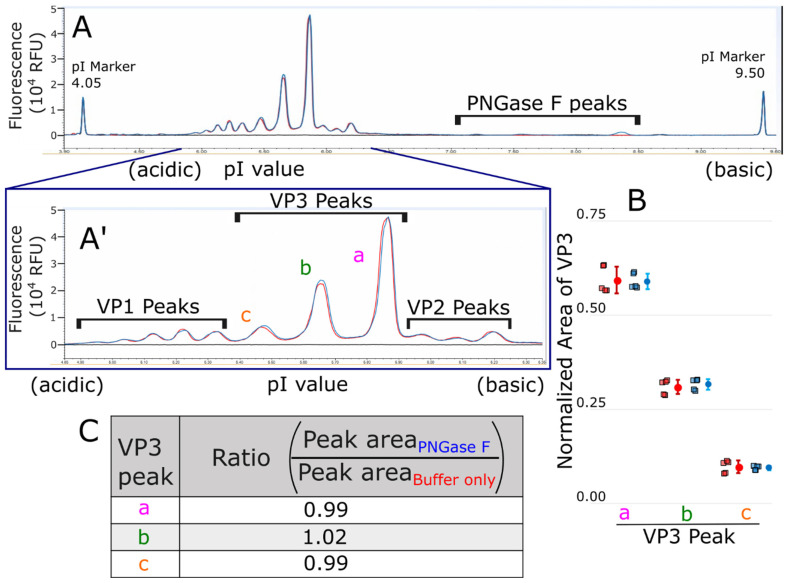
cIEF electropherograms of AAV9 capsids treated with PNGase F. (**A**) AAV9 capsids were treated with PNGase F (blue trace) or enzyme buffer (red trace) before denaturation and analysis by cIEF. Buffer only (black trace) refers to AAV9 formulation buffer with PNGase F enzyme buffer. (**A′**) Zoomed-in region of electropherogram showing VP-related peaks and their assignments. (**B**) Quantification of each one of three VP3 peaks, a, b, and c, relative to total VP3 peak area. Each individual value is shown as a square, and the mean is shown as a circle with error bars as one standard deviation. (**C**) Ratio of peak area of each VP3 peak comparing PNGase F treatment to buffer control.

We confirmed the N-glycosylation of AAV9 capsid proteins by using cIEF after the deglycosylation of the N-glycans from AAV9 capsid proteins using PNGase F. Figure 7A is the full view of the electropherogram of AAV9 capsid proteins and shows the PNGase F peaks, and Figure 7A’ is the zoomed-in electropherogram showing the AAV9 VP-related peaks based on the previously reported assignments [34]. A slight increase in the VP3 peak b area was detected after PNGase F treatment compared to a buffer-only control sample without PNGase F treatment (Figure 7A′), indicating that PNGase F treatment resulted in an enzymatic deamidation of VP3 proteins. This observation is consistent with the hypothesis that the AAV9 capsid is glycosylated. No changes in the peak areas of VP1 or VP2 were observed, possibly due to the low ratios of VP1 and VP2 to VP3 in an AAV capsid and the lower sensitivity of the cIEF method compared to mass spectrometry. 

To quantify VP3 deamidation by cIEF, for each injection, the peak areas of VP3 with no deamidation (pI ~ 5.8), VP3 with deamidation (pI ~ 5.6), and VP3 with more deamidation (pI ~ 5.4) [34] were summed to give the total VP3 peak area. The total VP3 peak area was then used to calculate the relative peak area for VP3 with no deamidation (VP3 peak a), with deamidation (VP3 peak b), and with more deamidation (VP3 peak c). The relative peak area for each VP3 peak, a, b, and c, was averaged over technical replicates; then, the average of the two biological replicates was calculated. This average was used to compare the ratio of each VP3 peak, a, b, and c, between the buffer-only control and PNGase F-treated capsids. A slight increase of about 2% in the peak area of VP3 with deamidation (pI about 5.6, VP3 peak b) was observed upon PNGase F treatment (Figure 7B,C), which is indicative of a low level of PNGase F-mediated deglycosylation.

**Figure 8:**
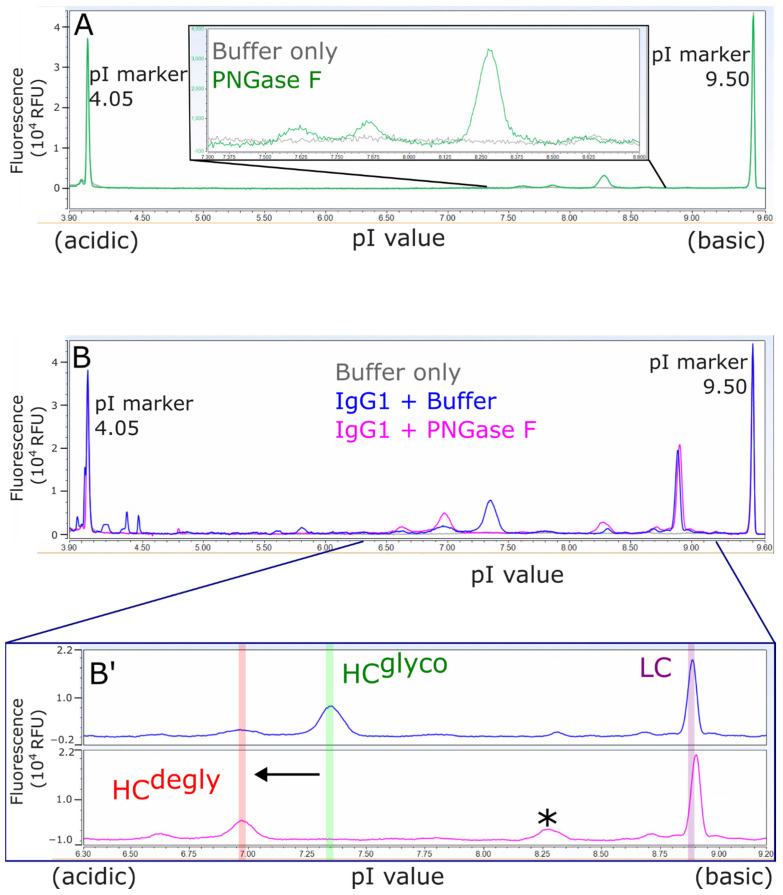
cIEF electropherograms of standard IgG1 monoclonal antibody as control. (**A**) Full view and inset of cIEF electropherograms of buffer only (protein formulation buffer and GlycoBuffer 2) or PNGase F. (**B**) Full view and (**B′**) zoomed-in region of cIEF electropherograms of human IgG1 treated with enzyme buffer or with PNGase F and subsequently reduced and denatured as described in Methods. In (**B′**), antibody light chain (LC), glycosylated heavy chain (HC^glyco^), and deglycosylated heavy chain (HC^degly^) are indicated. Asterisk (*) refers to a peak that has contribution from both antibody and PNGase F.

The standard IgG1 antibody was used as a control to ascertain the completion of deglycosylation. It was found that one hour of incubation with PNGase F at 37 °C was sufficient for the complete deglycosylation of the standard IgG1 antibody, as shown in Figure 8, and one hour was a sufficient compromise between maximizing the enzymatic deglycosylation and minimizing potential thermo-introduced deamidation. These conditions were used for the analysis of AAV9 capsid proteins.

## 5. Discussion

In contrast to nucleic acids and proteins, the biosynthesis of glycans is not directly template-driven; instead, glycosylation is a result of a complex network of metabolic and enzymatic reactions that are influenced by many factors, especially cell culture conditions, which greatly impact glycosylation forms for biotherapeutic products [24]. Oligosaccharyltransferase (OST) catalyzes the addition of N-glycans to the asparagine (N) in the consensus sequence motif, N-X-S/T [35]. However, the occupancy of glycosylation is affected by the neighboring amino acid sequence. A statistical analysis of the protein environment of N-glycosylation sites revealed that if the four amino acid residues before and one amino acid residue after the asparagine (N) in the N-X-S/T motif are aromatic amino acids (phenylalanine, tyrosine, or tryptophan), the probability of occupancy of the asparagine (N) in the N-X-S/T motif attached with N-glycans is higher; on the other hand, a negative charge around the asparagine (N) in the N-X-S/T motif may result in an unoccupied asparagine (N) in the N-X-S/T motif [36]. 

Based on the sequence of AAV9 capsid proteins, there are two acidic amino acids, glutamic acid (E) and aspartic acid (D) before N^14^LSE and one acidic amino acid, glutamic acid (E) after N^14^LS. This results in a negative charge around the asparagine (N) in the N-X-S/T motif and supports our observation that no N-glycans were found attached on N^14^LS. The four amino acid residues before and one amino acid residue after the asparagine (N) in the N-X-S/T motifs, N^262^ST, N^337^LT, N^452^GS, and N^497^NS, are not aromatic amino acids (phenylalanine, tyrosine, or tryptophan); therefore, low occupancies of N-glycans attached on N^262^ST, N^337^LT, and N^497^NS were observed. Asparagine (N) followed by glycine (G) forms the NG motif, which is the most potential deamidation site [11,12]. Deamidation may result in unoccupied N-glycans on N^452^GS. 

After the synthesis and transfer of an oligosaccharide precursor containing three glucose, nine mannose, and two N-acetylglucosamine sugars onto the asparagine residues in the consensus sequence motif (N-X-S/T) of proteins, N-glycans are processed in the endoplasmic reticulum by a series of glycan-processing enzymes and modified in the Golgi apparatus into complex glycans [37]. N-glycans are categorized into three forms: high mannose, hybrid, and complex. The hybrid and complex N-glycans were identified on AAV9 capsids (Table 2). When an N-glycan is removed by PNGase F from the side chain of asparagine (N) in the consensus sequence motif, the asparagine becomes aspartic acid (D), which results in a mass increase of 0.98402 Da [11,12] and the introduction of a negative charge [34]. Therefore, the deglycosylation of proteins can be detected by mass spectrometry based on the *m*/*z* ratio and cIEF based on charge.

O-linked glycosylation is the attachment of a sugar molecule to the oxygen atom of serine (S) or threonine (T) residues in a protein [38]. Proteins trafficked into the Golgi are most often O-glycosylated by N-acetylgalactosamine (GalNAc) transferase, which transfers a single GalNAc residue to the β-OH group of serine or threonine; there is no known consensus sequence for this enzyme. Therefore, in contrast to N-glycosylation sites, there is no general consensus protein sequence for O-glycosylation. O-glycosylation is not as complex as N-glycosylation; O-linked glycans usually have much simpler oligosaccharide structures than N-glycans. Several different sugars can be added to the serine or threonine, and they affect the protein in different ways by changing protein stability and regulating protein activity [39,40]. 

Mass spectrometry is a powerful tool for the determination of N- and O-glycosylation forms and sites. However, different mass spectrometers, software, database, and the fragmentation techniques used greatly impact the determination results. The mass spec data of glycopeptide mapping presented in this study were generated by using the higher-energy collisional dissociation (HCD) fragmentation technique at a collision energy of 30. Since the N-glycan diagnostic ion (HexNAc(1)) attached on N^497^(NS) was identified, in order to confirm if HCD may cause the in-source fragmentation of N-glycans linked on glycopeptides, the glycopeptide mapping of AAV9 capsid proteins was also carried out by using different fragmentation techniques. The fragmentation techniques employed include low-energy collision-induced dissociation (CID), step collision energies (SCEs) with multiple HCD collision energies of 15, 20, and 25 and scanned out in a single mass spectrum (sceHCD), electron-transfer dissociation (ETD), electron-transfer/collision-induced dissociation (ETciD), and electron-transfer/higher-energy collision dissociation (EThcD). No additional glycans were identified by using the aforementioned fragmentation techniques. 

The AAV9 capsids used for this study were generated from adherent cell culture. The glycopeptide mapping of AAV9 capsids processed from suspension cell cultures was also carried out. Similar to the results from the AAV9 capsids produced from adherent cell cultures, the N-glycans HexNAc(1) on N^497^(NS) and HexNAc(5)Hex(5) on N^337^(LT) and the O-glycans HexNAc(1) on S^499^ and HexNAc(2)Hex(1) on S^538^ were also observed on the AAV9 capsids processed from suspension cell cultures. 

Based on glycopeptide mapping for the identification of N- and O-glycosylation forms and sites, it was found that the occupancies of the N- and O-glycosylation of AAV9 capsids were low, resulting in low mass spec signals. To be confident about the results presented here, the N- and O-glycosylation forms and sites with poor y-ion and b-ion fragmentations or poor isotope plots by glycopeptide mapping are not reported. We also carried out an intact AAV9 capsid analysis using the RPLC-MS method [12], and multiple glycans attached on AAV9 capsids at low levels were identified. 

In contrast to glycopeptide mapping for the identification of both N-and O-glycosylation forms and sites, the released N-glycan analysis can only be used for the determination of released N-glycans. The released N-glycans may not only be obtained from the target AAV9 capsid proteins, but they may also come from impurities such as host proteins that are co-purified with the target proteins. Therefore, it may be necessary to characterize the N-glycosylation of target proteins using both glycopeptide mapping and released N-glycan analysis. Based on the labeled released N-glycan analysis for the determination of released N-glycans, the intensity of the released N-glycans from AAV9 capsids was more than 100 times lower than the intensity of the released N-glycans from human IgG1 (Figure 4), and 28 N-glycans at low abundances were identified (Table 2). The low occupancy of the N-glycosylation of AAV9 capsids results in the different identification of N-glycans of AAV9 capsids by glycopeptide mapping and released N-glycan analysis. 

Accurately characterizing N-glycan isomers is one of the limitations for both glycopeptide mapping by RPLC-MS/MS and released N-glycan analysis by HILIC LC-MS. HILIC LC-MS/MS would be able to determine the linkages present in N-glycan isomers if there are enough amount of released N-glycans. 

Based on the cIEF analysis, the deglycosylation of AAV9 capsids by PNGase F was about 2%, indicating that AAV9 capsids have low N-glycosylation levels (Figure 7). While the amount of deamidation due to the PNGase F-mediated deglycosylation detected by cIEF was low, this result was reproducible across the two biological replicates (a total of five technical replicates). Moreover, this result is consistent with the mass spectrometry results, which suggest that the occupancy of glycosylation in AAV capsids is low. In contrast to mass spectrometry, cIEF provides a low-resolution view of protein glycosylation, as the technique is unable to identify the glycosylation forms or the glycosite. Nevertheless, this approach is orthogonal to mass spectrometry and supports the hypothesis of AAV9 capsid glycosylation. 

Although glycosylation in non-enveloped viruses is thought to be less common [20], in addition to the previous reports on the glycosylation on AAV capsids [13,17,20], our study demonstrates that AAV9 capsids are sprinkled with sugars including N- and O-glycans at low occupancies. Altogether, our results may provide valuable information for a better understanding of AAV capsids and highlight the need to pay attention to glycosylation for AAV-based gene therapy development. 

## 6. Conclusions

AAV vectors have emerged as prominent gene delivery vehicles for gene therapy. The comprehensive characterization of AAV capsid proteins is necessary for AAV gene therapy. This study focused on the characterization of the glycosylation of AAV9 capsids generated from HEK293 cells. Using LC-MS/MS-based glycopeptide mapping and the released N-glycan analysis with HILIC LC-MS and HILIC LC-FLD, our study demonstrates that AAV9 capsids are sprinkled with sugars including N- and O-glycans. Furthermore, the N-glycosylation of AAV9 capsids was confirmed by the cIEF analysis. AAV capsid glycosylation may affect tissue tropism, vector assembly, cellular trafficking, and transduction efficiency. Although the glycosylated AAV9 capsids were in low abundance, we suggest paying attention to glycosylation for AAV-based gene therapy development. 

## Figures and Tables

**Table 1:** O-glycans identified by glycopeptide mapping.

O-Glycan	*m*/*z* Detected	Site
HexNAc(1)	203.0794	S^162^, S^469^, S^499^, S^586^, S^632^, T^241^
HexNAc(2)	406.1587	S^348^
HexNAc(2)Hex(1)	568.2116	S^538^, T^548^
HexNAc(1)Hex(1)NeuAc(1)	656.2276	T^339^, T^381^

**Table 2:** Identified released N-glycans from AAV9 capsid proteins.

No	Theoretical Mass	Experimental Mass	Mass Error (ppm)	Type	Composition	N-Glycan Structure
1	1912.7548	1912.7542	−0.3	Hybrid	HexNAc(3)Hex(6) *	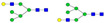
2	2099.8392	2099.8402	0.5	Complex	HexNAc(4)Hex(5)Fuc(1) *	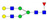
3	1879.7446	1879.7458	0.7	Complex	HexNAc(3)Hex(4)NeuAc(1) *	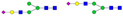
4	2033.7810	2033.7822	0.6	Complex	HexNAc(3)Hex(4)Fuc(1)NeuAc(1) *	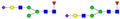
5	2041.7974	2041.7990	0.8	Hybrid	HexNAc(3)Hex(5)NeuAc(1) *	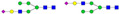
6	2244.8768	2244.8770	0.1	Complex	HexNAc(4)Hex(5)NeuAc(1) *	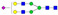
7	2390.9346	2390.9338	−0.3	Complex	HexNAc(4)Hex(5)Fuc(1)NeuAc(1) *	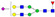
8	2756.0668	2756.0670	0.1	Complex	HexNAc(5)Hex(6)Fuc(1)NeuAc(1) *	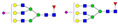
9	3122.2062	3122.2080	0.6	Complex	HexNAc(6)Hex(7)Fuc(1)NeuAc(1) *	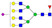
10	2682.0300	2682.0312	0.5	Complex	HexNAc(4)Hex(5)Fuc(1)NeuAc(2) *	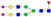
11	2349.9082	2349.9082	0.0	Hybrid	HexNAc(3)Hex(6)Fuc(1)NeuAc(1)	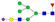
12	2203.4502	2203.4506	0.2	Hybrid	HexNAc(3)Hex(6)NeuAc(1)	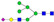
13	2534.7722	2534.7736	0.6	Complex	HexNAc(4)Hex(5)NeuAc(2)	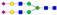
14	2286.9238	2286.9238	0.0	Complex	HexNAc(5)Hex(4)Fuc(2)	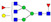
15	2157.0608	2157.0606	−0.1	Complex	HexNAc(5)Hex(5)	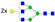
16	2301.7186	2301.7198	0.6	Complex	HexNAc(5)Hex(5)Fuc(1)	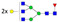
17	2463.1714	2463.1736	0.9	Complex	HexNAc(5)Hex(6)Fuc(1)	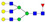
18	2901.8115	2901.8118	0.1	Complex	HexNAc(5)Hex(6)NeuAc(2)	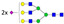
19	3047.8695	3047.8698	0.1	Complex	HexNAc(5)Hex(6)Fuc(1)NeuAc(2)	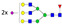
20	3189.3072	3189.3111	1.3	Complex	HexNAc(5)Hex(6)NeuAc(3)	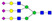
21	3338.9649	3338.9652	0.1	Complex	HexNAc(5)Hex(6)Fuc(1)NeuAc(3)	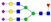
22	3245.2488	3245.2551	2.0	Complex	HexNAc(6)Hex(6)Fuc(1)NeuAc(2)	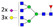
23	2960.2485	2960.2611	4.3	Complex	HexNAc(6)Hex(7)NeuAc(1)	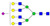
24	3268.1439	3268.1427	−0.3	Complex	HexNAc(6)Hex(7)NeuAc(2)	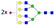
25	3410.6016	3410.6046	0.9	Complex	HexNAc(6)Hex(7)Fuc(1)NeuAc(2)	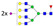
26	3599.1393	3599.1261	−3.6	Complex	HexNAc(6)Hex(7)NeuAc(3)	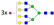
27	3704.0973	3704.0976	0.1	Complex	HexNAc(6)Hex(7)Fuc(1)NeuAc(3)	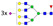
28	3995.4927	3995.4927	0.0	Complex	HexNAc(6)Hex(7)Fuc(1)NeuAc(4)	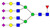

Note: * indicates the N-glycans that were identified in AAV8 [19].

## Data Availability

All the data generated in this study are available in the manuscript.
